# Photo-enhanced Aqueous Solubilization of an Azo-compound

**DOI:** 10.1038/s41598-017-06947-w

**Published:** 2017-07-31

**Authors:** Minoru Ishikawa, Takuya Ohzono, Takao Yamaguchi, Yasuo Norikane

**Affiliations:** 1 Institute of Molecular and Cellular Biosciences, The University of Tokyo, 1-1-1 Yayoi, Bunkyo-ku, Tokyo 113-0032 Japan; 2 Research Institute for Sustainable Chemistry, National Institute of Advanced Industrial Science and Technology (AIST), 1-1-1 Higashi, Tsukuba, Ibaraki 305-8565 Japan; 3 Electronics and Photonics Research Institute, National Institute of Advanced Industrial Science and Technology (AIST), 1-1-1 Higashi, Tsukuba, Ibaraki 305-8565 Japan

## Abstract

We previously showed that disruption of intermolecular interactions, e.g., by lowering the molecular planarity and/or introducing bent structures, improves the aqueous solubility of compounds, and based upon that work, we hypothesized that azobenzene *trans*-to-*cis* photoswitching could also be utilized to enhance the aqueous solubility of compounds. Here, we demonstrate that UV/visible light irradiation can reversibly switch the aqueous solubilization of an anti-cancer candidate drug, a low-molecular-weight kinase inhibitor bearing an azobenzene moiety. The increase of solubilization associated with UV-induced *trans*-to*-cis* conversion may have clinical relevance, because the time-scale of thermal *cis*-to-*trans* reversion at 37 °C is longer than that of oral absorption.

## Introduction

Aqueous solubility is a key physicochemical property of molecules, influencing ease of synthesis and purification, chemical-biological properties, functions of materials, effect on the environment, and so on^1–3^. For example, from the viewpoint of drug development, candidate compounds possessing sufficient aqueous solubility are considered to offer a relatively low risk of failure^4–7^.

The principle of solvation is “like dissolves like”, reflecting the fact that organic compounds generally dissolve in organic solvents, but not in water. Thus, enhancement of the aqueous solubility of organic compounds is generally challenging. Decrease of hydrophobicity (the common logarithm of partition coefficient: Log*P*_ow_) by chemical modification, i.e., introduction of hydrophilic group(s) into compounds, is a classical and general strategy for improving aqueous solubility. On the other hand, the solubility of a solid solute in water is also dependent on the crystal packing of the solute^4^ suggesting the existence of another principle of solvation. Compounds that are insoluble due to crystal packing interaction have been called “brick dust”^4^. Therefore, an alternative strategy to improve aqueous solubility by molecular modification is to weaken the intermolecular interaction of the compound. But, early studies to elucidate how the intermolecular interaction of organic compounds affects solubility in water were quite limited^6, 8^, although there is an old rule of thumb that organic compounds possessing weaker intermolecular interaction tend to show higher solubility in organic solvents or in mixtures of organic solvent and water. On the other hand, during the last decade, several strategies for improving the aqueous solubility of pharmaceutical compounds by disrupting intermolecular interactions have been developed^6–9^. We have shown that these strategies for disrupting intermolecular interactions can increase the aqueous solubility of molecules even if their hydrophobicity is concomitantly increased^6, 8, 9^. It is noteworthy that the “brick dust” principle appears to have a greater influence than the “like dissolves like” principle on the aqueous solubility of various compounds.

Azobenzenes are a group of photoswitchable molecular machines that can exist in either *trans* or *cis* form. *trans*-Azobenzenes have planar conformations that are thermodynamically more stable, and they can be generated by visible light irradiation or spontaneously by thermal isomerization. On the other hand, irradiation of azobenzenes with UV light generates the *cis* isomers. Azobenzene photoswitches have been utilized for organic synthesis^10, 11^, functional materials including self-healing materials^12^, adhesives^13, 14^, photoresists^15^ and optical materials^12, 16, 17^. In these applications, they have been employed mainly in solid/liquid states or as solutions in organic solvents. Currently, however, there is increasing interest in photoswitches that work in aqueous solution, for example, for modulation of biological activities^18–20^, for bioimaging^21^ or for phase transition^22^.

**Figure 1:**
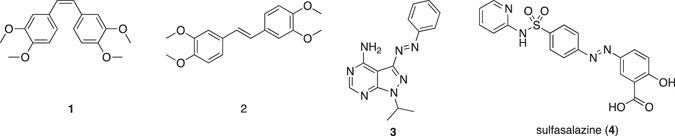
Chemical structures of compounds used in this study.

*cis*-Azobenzene adopts a bent conformation with its phenyl rings twisted about 55° out of the plane from the azo group^18^. Thus, *cis*-azobenzenes may exhibit weaker intermolecular interactions than their *trans* isomers. Indeed, several groups^13^, including ours^23, 24^, have reported that azobenzenes melt under UV irradiation. Therefore, we hypothesized that *cis*-azobenzenes would possess better aqueous solubilization than their *trans* isomers as a result of the weaker intermolecular interactions. Some evidence of photo-induced solubilization change has already been reported for azobenzenes^11, 25, 26^ or azo-poly(glutamic acid), azo-poly(ornithine) and azo-poly(diaminopropanoic acid)^27–30^. However, because of the essentially hydrophobic character of azobenzene, those studies were limited to turbidity measurements in organic solvents^11, 25, 26^ or organic solvent/water mixtures^27–30^. On the other hand, water has many advantages as a solvent, such as environmentally friendly and non-inflammable character, and is irreplaceable in medical and biological applications. It should also be noted that the physical properties of smaller molecules differ from those of polymers, which possess much stronger intermolecular forces and higher melting points; indeed, photoswitching of the azo-polymers is associated with higher-order structural change^27^. The photo-induced change of aqueous solubilization of low-molecular-weight compounds bearing an azobenzene moiety, in the absence of organic solvent, remains an interesting lacuna in current knowledge. Moreover, the mechanism of solubilization change of azobenzenes, including the kinetic process, in response to light irradiation remains unclear. Therefore, direct, systematic and quantitative examination of the photo-induced change of aqueous solubilization of low-molecular-weight azobenzenes is required. In this study, we investigated the effect of light irradiation on the aqueous solubilization of a RET kinase inhibitor **3**, a low-molecular-weight anti-cancer candidate drug bearing an azobenzene moiety (Fig. 1). Importantly, we found that exposure to UV light increased the aqueous solubilization by up to 20-fold. Detailed studies to elucidate the mechanism revealed that UV irradiation promotes transformation of the *trans* isomer to the *cis* isomer, not only in solution, but also on the surface of suspended solid particles. In this report, we use the term “solubilization” but not “solubility” because the irradiation conditions are neither the closed systems nor the equilibrium conditions.

## Results and Discussion

### Evaluation of trans- and cis-stilbenes as model compounds of azobenzenes

First, we synthesized *cis*- and *trans*-stilbene analogs **1** and **2** as models for *cis-* and *trans*-azobenzenes, respectively (Fig. 1, Fig. S1). As expected, *cis*-**1** was 14 times more soluble than *trans*-**2** in phosphate buffer (Table S1). To confirm the putative mechanism of the higher solubility of the *cis* isomer, the melting point and Log*P*_ow_ were examined as parameters of intermolecular interaction and hydrophobicity, respectively^4^. The order of lower melting points (*cis* < *trans*) was the same as the order of higher aqueous solubility (*cis* > *trans*). As for hydrophobicity, the Log*P*_ow_ values of **1** and **2** were almost the same. Overall, these results support the idea that the higher aqueous solubility of *cis*-**1** than *trans*-**2** is due not to a decrease of hydrophobicity, but rather to a loosening of intermolecular interaction. Therefore, the bent and twisted structure of *cis*-azobenzenes may result in greater aqueous solubility.

### Confirmation of *cis-trans* isomerization of a bioactive azobenzene 3

To confirm the potential usefulness of this strategy for improving aqueous solubility, we focused on kinase inhibitor **3** ^31^ (Fig. S2) as representative of pharmaceutical compounds, because the 518 human protein kinases include important drug targets, and the molecular structures of many kinase inhibitors tend to be rather similar. We confirmed that a solution of **3** in water containing 1% MeOH showed reversible light-induced *trans-cis* photoswitching (Figs S3, S4 and Table S2) in accordance with the previous report^15^.

### Aqueous solubilization under irradiation with UV light

**Figure 2:**
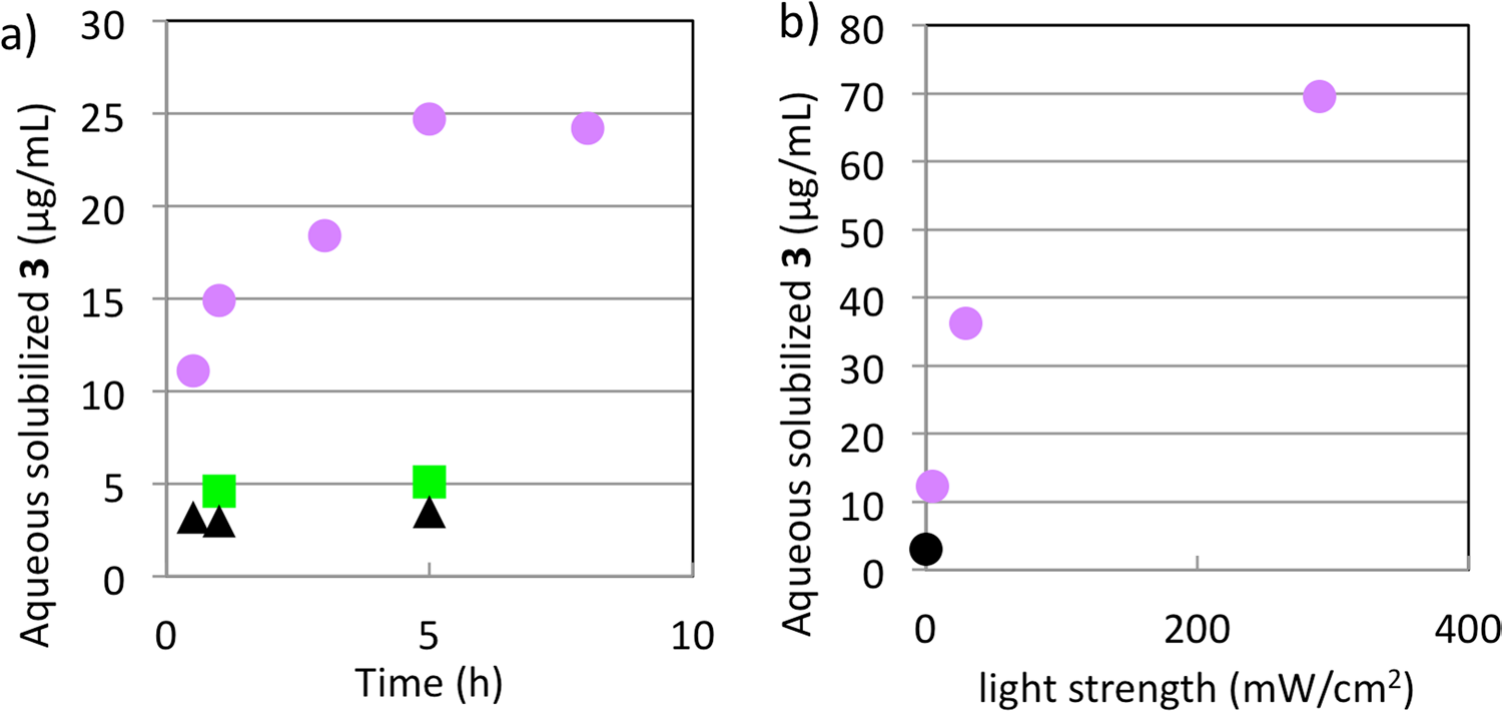
Aqueous solubilization of **3**. (**a**) Solubilization under UV irradiation (magenta circles), under visible light irradiation (green squares), and in the dark (black triangles) at 37 °C. UV irradiation was performed with a mercury lamp (0.5 mW/cm^2^). (**b**) Solubilization of **3** under UV irradiation at various intensity levels. Sample irradiation was conducted using 365 nm LED light at 37 °C for 1 h.

Next, the solubility of **3** in phosphate buffer was evaluated. In the dark, the aqueous solubility of **3** after 0.5, 1 and 5 h at 37 °C was approximately 3 μg/mL (Fig. 2a and Table S3), indicating that **3** reached solubility equilibrium within 0.5 h. In contrast, the aqueous solubilization of **3** under UV irradiation continued to increase, reaching a plateau after 5 hours. Thus, UV irradiation effectively led to increased aqueous solubilization compared with the dark condition (3.5-fold at 0.5 h, 5.0-fold at 1 h, and 7.1-fold at 5 h). Under white-light irradiation, the solubilization of **3** after 1 and 5 h was approximately 5 μg/mL. The values of the *cis* ratio of **3** in the solution after 1 h of UV irradiation, 1 h of visible light irradiation, and in the dark were 80%, 25% and 4%, respectively, and remained roughly constant during the evaluation period (Table S3). The order of conditions giving higher aqueous solubility (UV light >visible light >dark) was the same as the order giving higher *cis* ratio in solution. This result indicates that *trans* → *cis* isomerization is important for higher aqueous solubility of **3**.

### Influence of UV wavelength and light intensity on aqueous solubilization

To validate the idea that a higher *cis* ratio in solution is important for higher aqueous solubilization of **3**, we investigated the aqueous solubilization of **3** under irradiation at various wavelengths in the range of 355–540 nm through filters of various bandwidths for 1 h (Fig. S5 and Table S4). We found that both the *cis* ratio of **3** contained in the solution after irradiation and the solubilization of **3** were dependent on wavelength, and were increased as light of shorter wavelengths was employed. This result supports the conclusions that the choice of illuminating wavelength determines the ratio of the *cis* form generated, and that the ratio of the *cis* form is the primary determinant of the aqueous solubilization. We could not completely convert the *trans* form to *cis*, and irradiation seemed to produce a photostationary state with a maximum of 90% *cis* (irradiation at 380 nm) or 93% *trans* (irradiation at 535 nm). Possible reasons for this would be that the absorption spectra of the *trans* and *cis* isomers overlap substantially^18^, and/or that slight isomerization occurred during the filtration and HPLC evaluation as a result of exposure to ambient light.

Irradiation through a filter with wider bandwidth tended to increase the solubilization of **3** (Fig. S5: 355 nm (bandwidth: 40 nm) and 380 nm (bandwidth: 10 nm), and 535 nm (bandwidth: 25 nm) and 540 nm (bandwidth: 8 nm)). A possible reason for this is that higher irradiance with wider bandwidth filters generated a higher ratio of the *cis* form. Therefore, we next checked whether light intensity itself affects the solubilization of **3**. The solubilization of **3** increased as the UV intensity was increased, and the maximum irradiation intensity examined (290 mW/cm^2^) led to a 23-fold increase of aqueous solubilization compared with the dark condition (Fig. 2b and Table S5). The *cis* ratio of **3** contained in the solution after UV irradiation remained roughly constant (90%), independent of the light intensity (Table S5). Reversible change of the aqueous solubilization of **3** under UV/visible light irradiation was also confirmed (Fig. S6 and Table S6).

### Possible generation of *cis* solid under UV irradiation

**Figure 3:**
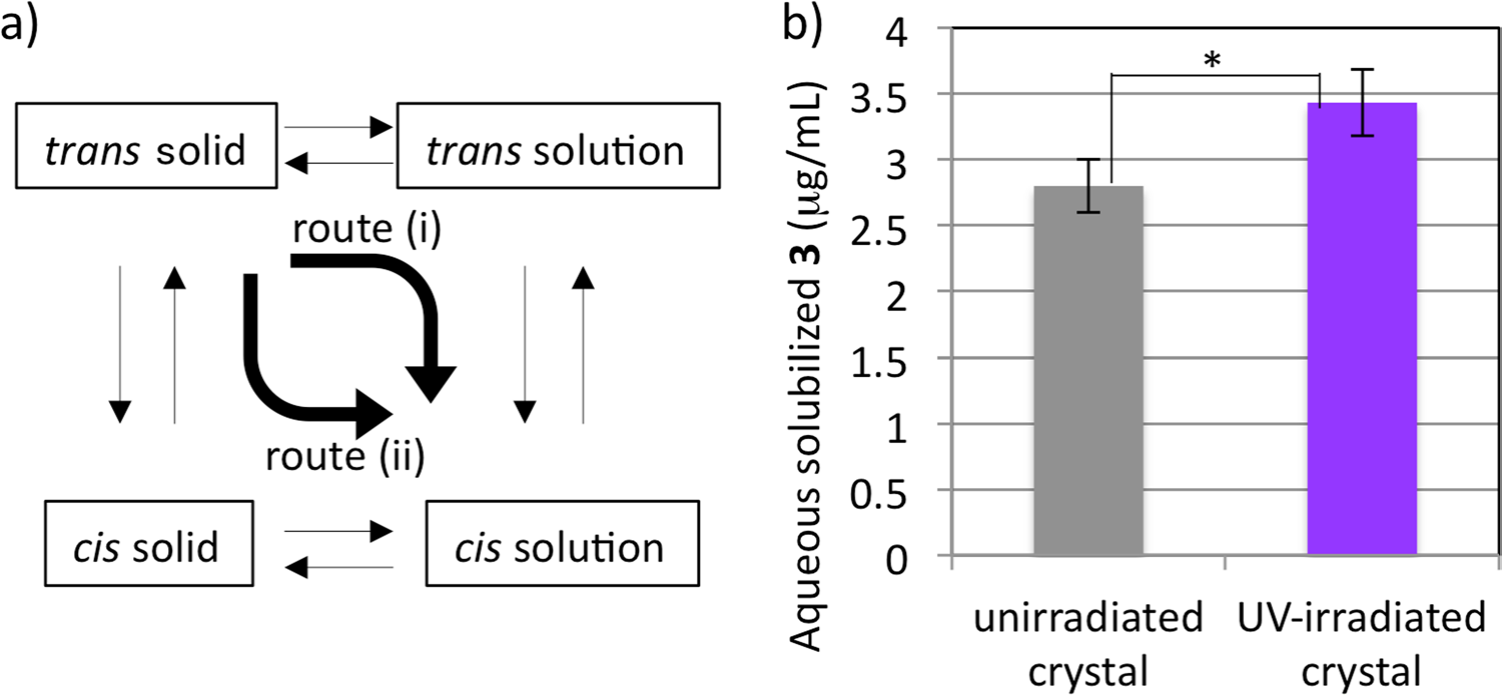
(**a**) Possible equilibria and routes for generating *cis*-**3** in solution. (**b**) Kinetics of aqueous dissolution of UV-irradiated crystals of **3**, measured in the dark. Crystals of **3** were irradiated with 365 nm LED light (344 mW/cm^2^) at 37 °C for 3 h. The UV-irradiated crystals and unirradiated crystals were each shaken with phosphate buffer in the dark at 37 °C for 1 h. Data are the mean ± SD of three individual experiments. The asterisk indicates a significant difference (*p < 0.05) from the unirradiated crystals according to the unpaired, two-sided Student’s *t*-test.

At the photostationary state under UV irradiation, the equilibria shown in Fig. 3a should be in balance. Here, we are interested in the transient process, i.e., how *cis*-**3** in solution increases when UV irradiation is started. There are two possible routes for generating *cis*-**3** in solution from *trans*-**3** in the solid state: route (i) and (ii). Route (i) is clearly possible, because the process *trans* solution → *cis* solution is easily driven by UV irradiation (Fig. S3a). The remaining question is whether *cis* isomerization of solid **3** is induced under UV irradiation, that is, whether route (ii) also operates. To answer this question, we investigated the aqueous dissolution in the dark of UV-irradiated crystals of **3**. The UV-irradiated crystals were clearly more soluble than the unirradiated crystals (Fig. 3b and Table S7). In addition, the *cis* ratio of UV-irradiated crystals contained in the solution was higher than that of unirradiated crystals (14% vs 4% on average, Table S7). These results strongly support the idea that UV irradiation of crystals of *trans*-**3** can induce isomerization to solid *cis*-**3** to some extent, presumably near the crystal surface. Therefore, we conclude that routes (i) and (ii) both operate upon UV irradiation.

Increased local temperature induced by UV irradiation might contribute in part to the increased solubilization of **3** in the experiments of Figs 2a,b, S5, and S6. We therefore confirmed the aqueous solubility at 46 °C, the temperature of upper limit of our equipment. The solubility at 46 °C (3.5 μg/mL after 1 h and 3.2 μg/mL after 24 h) was slightly higher than that at 37 °C (Fig. 2a), but the rise in temperature seemed to have little impact on the aqueous solubility of **3**. Meanwhile, we observed increased dissolution of pre-UV-irradiated crystals of **3** in the absence of irradiating UV light during dissolution evaluation (Fig. 3b). It is noteworthy that there would be no increase of local temperature in this case, so these results are consistent with our hypothesis that photo-induced conformational change induced on the crystals contributes predominantly to the higher aqueous solubilization of UV-irradiated **3**.

### Dissolution of *cis* isomer from solid surface: route (ii)

**Figure 4:**
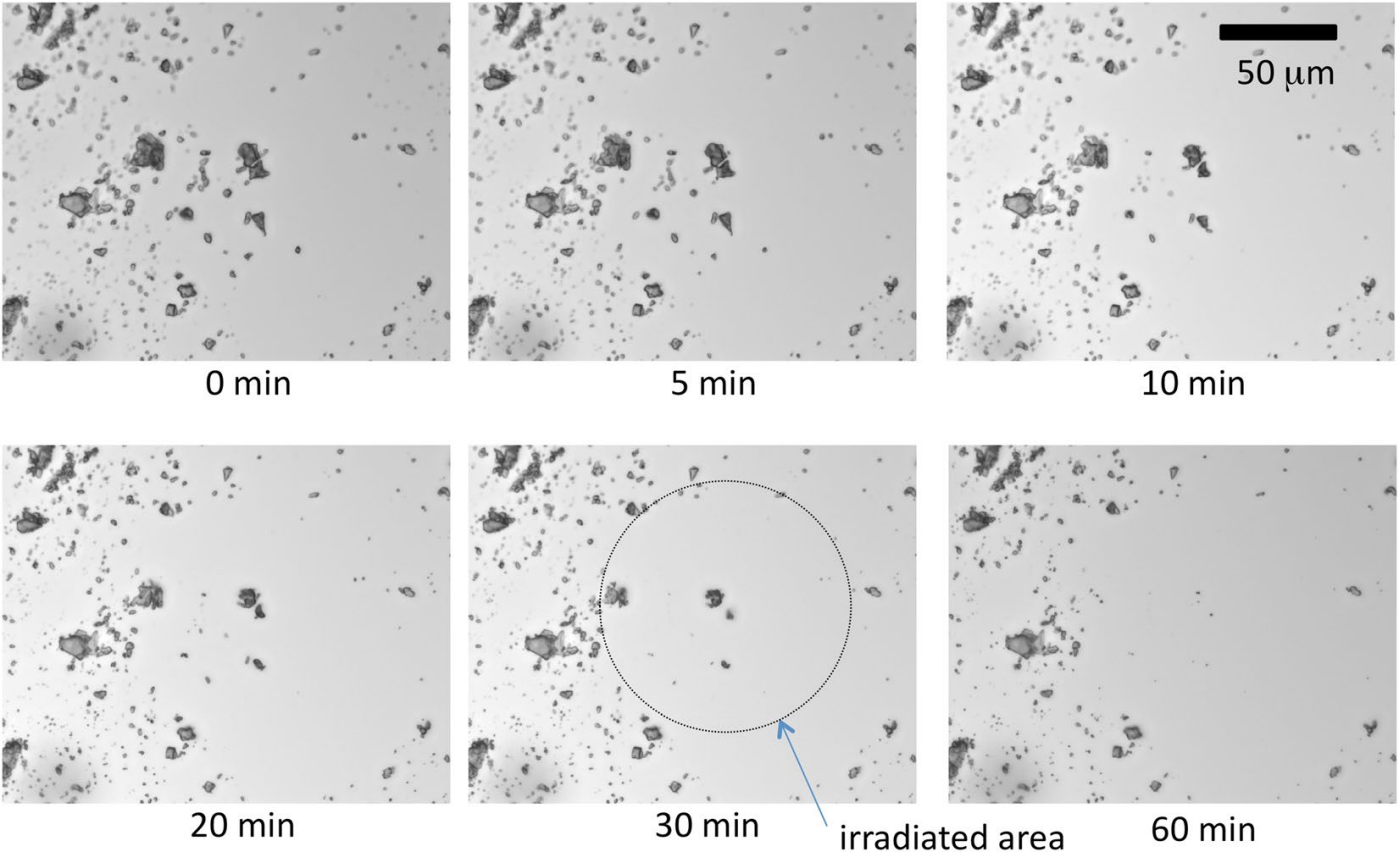
Bright-field microscopy images of crystals of **3** in water before and after UV irradiation (330–385 nm, 290 mW/cm^2^) for 5, 10, 20, 30 and 60 min at room temperature. The region between the slide glass and cover glass of the sample shown in Fig. S8 was filled with water before this observation.

To confirm the operation of route (ii) under UV irradiation, the microscopic morphology of the crystals was investigated. Firstly, as a reference experiment, dry crystals were observed under an optical microscope to evaluate the effect of UV irradiation on their optical features. Bright-field and polarized images showed no marked change after UV irradiation (Figs S7 and S8). Thus, we concluded that the crystalline particles remain essentially unchanged at the micrometer scale; no overall melting was induced by UV irradiation. Next, crystals were placed between a glass slide and a cover glass with the cell gap of 27 ± 7 μm, the space between the glasses was filled with water, and the UV-irradiated suspension was observed under the optical microscope. Under these conditions, only crystals located in the UV-irradiated region diminished and disappeared (Fig. 4 and Movie S1). If the major mechanism of increased solubilization is route (i), the solubilized *cis*-**3** should be produced via photo-isomerization only from *trans*-**3** that is supplied from the crystalline *trans*-**3** as shown in Fig. 3a. Since the produced concentration differences between the irradiated and non-irradiated areas are partially damped via diffusion, the local concentration of *trans*-**3** around the irradiated area would also drop. Thus, crystals outside of the UV-irradiated region would also disappear or shrink to supply *trans*-**3** to the liquid phase, but in fact this was not observed. (Assuming the diffusion constant of a general solute in water *D* ~ 10^−9^ m^2^s^−1^, within the present experimental timescale *t* ~ 10^3^ s, the molecules would diffuse over a distance of *L* ~ (*Dt*)^1/2^ ~ 1 mm. Thus, the effect of the decreased concentration of *trans*-**3** in the UV-irradiated area would influence the outer region in the optical images (Fig. 4); the concentration difference would be largely diminished over a scale of tens of micrometers, which is the scale of the images shown in Fig. 4). Consequently, the present direct observation of crystals suggests that dissolution of the crystals mainly proceeds via route (ii) and only molecules on the surface on the irradiated crystals would be photo-isomerized to *cis* form.

### Aqueous solubilization of sulfasalazine (4) under UV irradiation

Sulfasalazine (**4**) (Fig. 1), which is used in the treatment of rheumatoid arthritis, is another pharmaceutical compound bearing an azobenzene moiety with push-pull systems, which cause very fast thermal isomerization^3^. Therefore, we also examined **4** in order to throw further light on the results obtained with **3**. UV-visible spectroscopy revealed no isomerization of **4** after UV irradiation for 30 min (Fig. S9). Further, the aqueous solubilization of **4** was evaluated under UV irradiation (0.5 mW/cm^2^) and in the dark at 37 °C for 5 h (Table S8), and was essentially the same in each case. More intense UV irradiation (344 mW/cm^2^) resulted in a slight increase (1.4-fold) of solubilization. Compound **3** possessing a smaller molar extinction coefficient ε (Logε at 365 nm: 4.085) showed significantly increased aqueous solubilization under UV irradiation (Fig. 2a: 7-fold and Fig. 2b: 23-fold), whereas **4** possessing the higher ε (Logε at 365 nm: 4.458) showed only a 1.4-fold increase of solubilization (Table S8). This result again suggests that increased local temperature is not a major mechanism of the increased solubilization of **3** in response to UV irradiation. By contrast, the slight increase (1.4-fold) of solubilization of **4** by UV irradiation might have been due to increased local temperature, and/or by (short-term) isomerization to the *cis* isomer.

### Possible application for optical control of aqueous solubilization of medicinal drugs

A prodrug is defined as a compound that is converted within the body into a bioactive drug after administration. Among several types of prodrugs, water-soluble prodrugs synthesized by adding an ionizable pro-moiety to the parent molecule have been developed. Water-soluble prodrugs are useful for increasing not only solubility but also bioavailability after oral dosing, when this is limited by slow dissolution. The use of photocontrolled prodrugs to manage aqueous solubility is a novel concept. For example, the *cis* isomer of a water-insoluble *trans*-azobenzene-bearing drug might be formed by UV irradiation and administered to obtain increased aqueous solubility and/or bioavailability, and then would subsequently be converted to the bioactive *trans* isomer within the body by thermal isomerization. Actually, the half-life of thermal isomerization of *cis*-**3** at 37 °C was estimated as 5.6 hours (Fig. S10 and Table S9), which is similar to the time-scale of oral absorption. A 20-fold increase of solubility would be valuable in medicinal chemistry^8^.

## Conclusions

Here, we showed that the aqueous solubilization of a small molecule bearing an azobenzene moiety could be controlled reversibly by irradiation with UV and visible light. The aqueous solubilization of **3** varied depending on the UV irradiation wavelength and intensity. The solubilization of compound **3** in phosphate buffer was increased by up to 20-fold by exposure to UV irradiation, compared to that without irradiation. Detailed studies of the mechanism of the enhancement of aqueous solubilization of **3** revealed that UV irradiation promotes transformation of *trans* to *cis* isomer not only in solution, but also on the surface of suspended solid particles. Our results indicate that this photo-induced change to *cis* molecular conformation is the predominant contributor to the higher aqueous solubilization, as we had anticipated on the basis of our previously developed strategies for tuning aqueous solubility. The increase of solubilization associated with UV-induced *trans*-to*-cis* conversion may have practical pharmaceutical relevance, because the time-scale of thermal *cis*-to-*trans* reversion at 37 °C is longer than or similar to that of oral absorption.

## Electronic supplementary material

Supplementary Information

movie S1
