# Supplementary material for: Photo-enhanced Aqueous Solubilization of an Azo-compound
Source: Sci Rep. 2017 Jul 31;7:6909. doi: 10.1038/s41598-017-06947-w (PMC5537305; doi:10.1038/s41598-017-06947-w)
Supplement: Supplementary file 1 — Supplementary Information [file 41598_2017_6947_MOESM1_ESM.pdf]

Supplementary Information

Photo-enhanced Aqueous Solubilization of an Azo-compound

Minoru Ishikawa,[1]* Takuya Ohzono,[2] Takao Yamaguchi,[1] Yasuo Norikane[3]

[1] Institute of Molecular and Cellular Biosciences, The University of Tokyo, 1-1-1 Yayoi, Bunkyo-ku, Tokyo 113-0032, Japan

[2] Research Institute for Sustainable Chemistry, National Institute of Advanced Industrial Science and Technology (AIST), 1-1-1 Higashi, Tsukuba, Ibaraki 305-8565, Japan

[3] Electronics and Photonics Research Institute, National Institute of Advanced Industrial Science and Technology (AIST), 1-1-1 Higashi, Tsukuba, Ibaraki 305-8565, Japan

Corresponding author: Minoru ISHIKAWA

Institute of Molecular and Cellular Biosciences, The University of Tokyo

1-1-1, Yayoi, Bunkyo-ku, Tokyo, 113-0032, Japan

Tel.: +81 3 5841 7853; fax: +81 3 5841 8495

E-mail address: m-ishikawa@iam.u-tokyo.ac.jp

Contents

Figures S1-S10	Page 3
Tables S1-S9	Page 9
Movie S1	Page 13
Experimental Section	Page 14

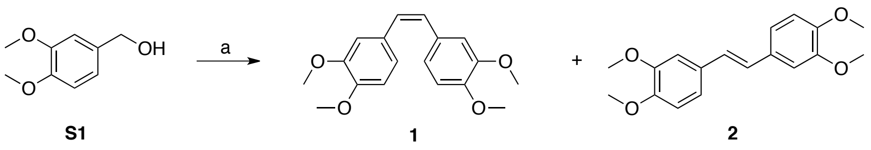

Figure S1. Reagents and conditions: a) i) NBS, PPh_3, toluene, reflux, ii) 3,4-dimethoxybenzaldehyde, K_2CO_3, toluene, reflux.

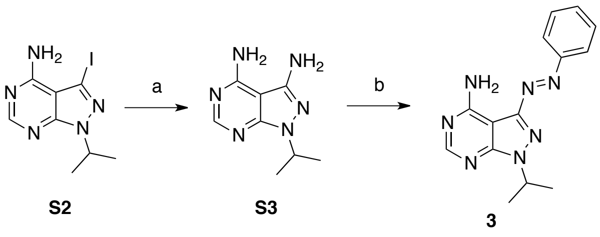

Figure S2. Reagents and conditions: a) CuI, L-proline, K_2CO_3, NH_4OH, DMSO, 60 ºC, 53%, b) PhNO, AcOH, 60 ºC, 65%.

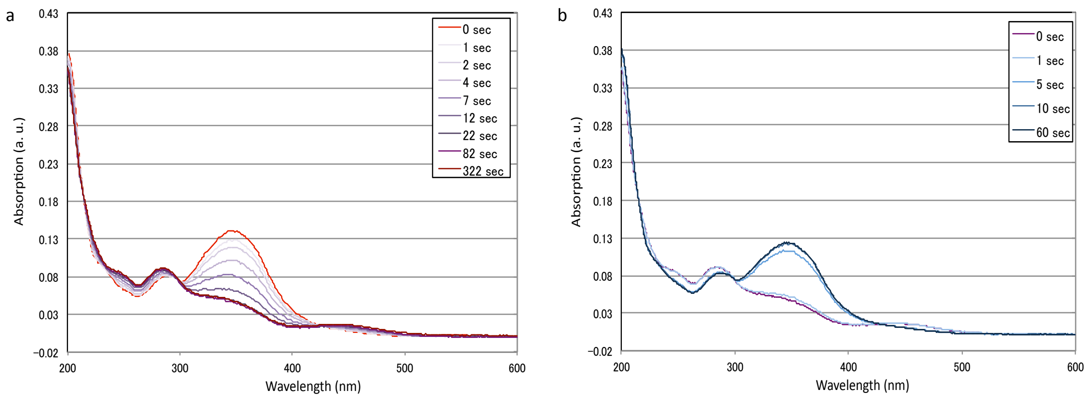

Figure S3. Photoinduced isomerization of **3** (10 μM) in a mixture of water and MeOH (1%). a) A solution of *trans*-**3** was successively irradiated with a 365 nm mercury lamp (0.5 mW/cm^2) for 1 s, 2 s, 4 s, 7 s, 12 s, 22 s, 82 s and 322 s. b) Isomerized *cis*-**3** was successively irradiated with white light (68 mW/cm^2 at 460 nm) for 1 s, 5 s, 10 s, and 60 s.

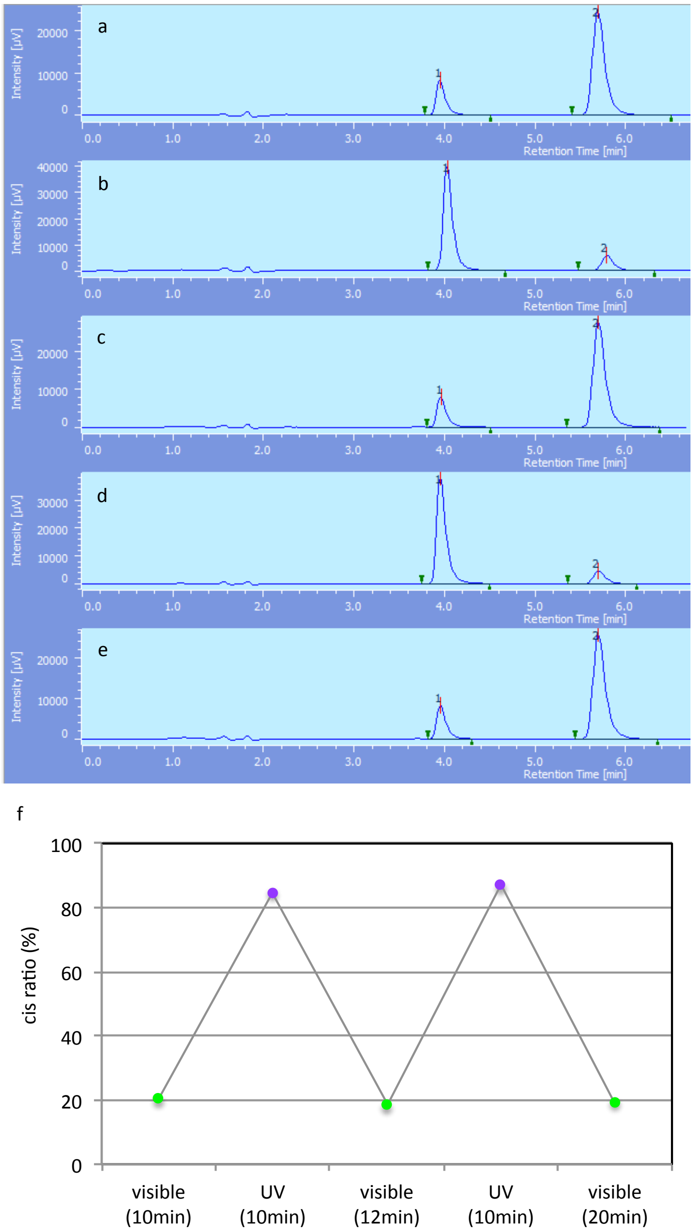

Figure S4. Photoinduced isomerization of **3** (10 µM) in a mixture of water and MeOH (1%). Dissolved *trans*-**3** was successively exposed to white light (68 mW/cm^2 at 460 nm) and to a 365 nm mercury lamp (0.5 mW/cm^2) for 10 min, 10 min, 12 min, 10 min, and 20 min. a-e) HPLC profile of irradiated solution. f) *cis* ratio of irradiated solution. The photostationary distributions reached under the UV lamp and under white light were determined by HPLC to be approximately 85% *cis* form and approximately 20% *cis* form, respectively.

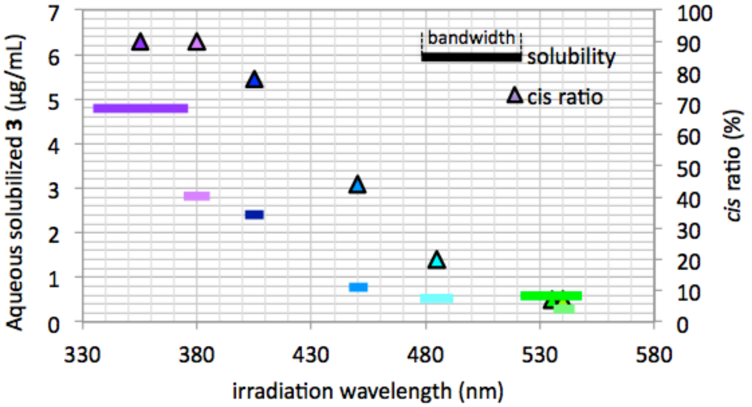

Figure S5. Aqueous solubilization (bars) and *cis* ratio (triangles) of **3** under irradiation at various wavelengths. Illumination was performed with a xenon flashlamp using repeated cycles of irradiation and shaking (5000 flashes, then shaking for 10 sec) through filters of various bandwidths (indicated by the width of the bar) at room temperature (20-22 ºC) for 1 h. *Cis* ratio in the aqueous solution was evaluated from the peak areas in HPLC immediately after filtration.

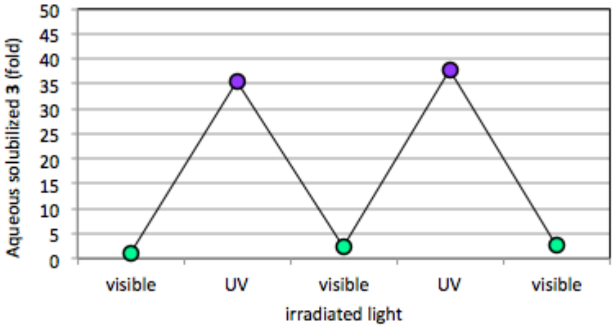

Figure S6. Reversibility of aqueous solubilization changes of **3** under successive UV (magenta) /visible (green) light irradiations. Sample irradiations were conducted with white light through a filter cutting off wavelengths below 560 nm, or with 365 nm LED light (344 mW/cm^2) at 37 ºC for 1 h. UV irradiation after an initial visible light irradiation caused a 35.5-fold increase of aqueous solubilization. Subsequent visible light irradiation caused the aqueous solubilization to revert almost to the original level (2.3-fold compared with the initial visible irradiation condition). Subsequent UV and visible light irradiations also led to reversible changes of aqueous solubilization.

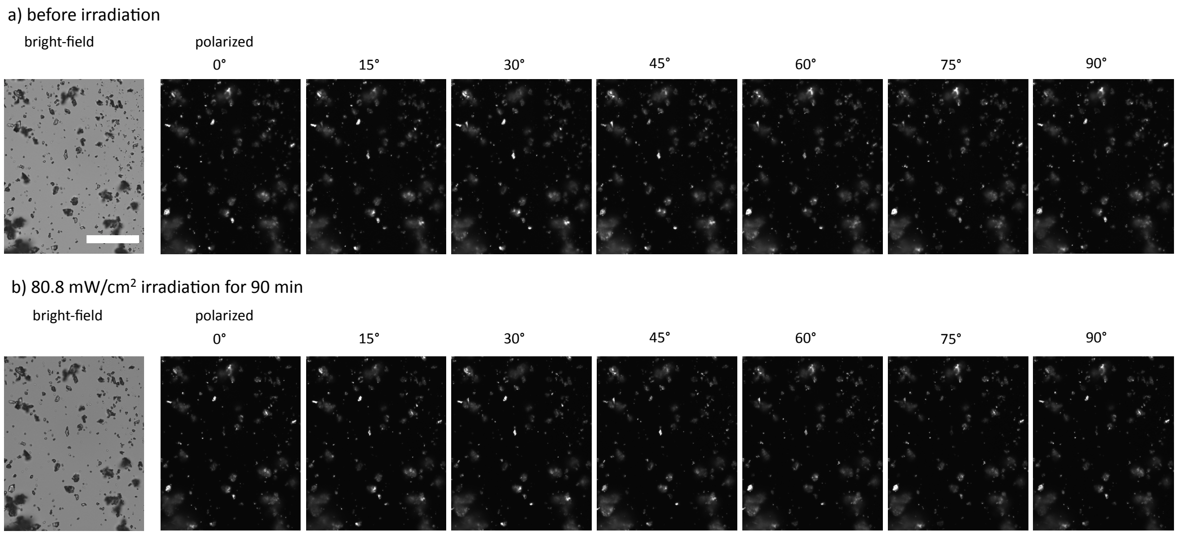

Figure S7. Bright-field microscopy images (leftmost), and polarized microscopy images under crossed polarizers at different angles of sample rotation (images with dark backgrounds) before irradiation (a) and after UV irradiation (b) of crystals of **3** in a dry condition. As the sample is rotated, the brightness of the particles changes and an extinction position exists, suggesting that the particles are in the crystalline stte. UV irradiation (330-385 nm, 81 mW/cm^2) was done for 90 min at room temperature. The calibration bar represents 50 μm.

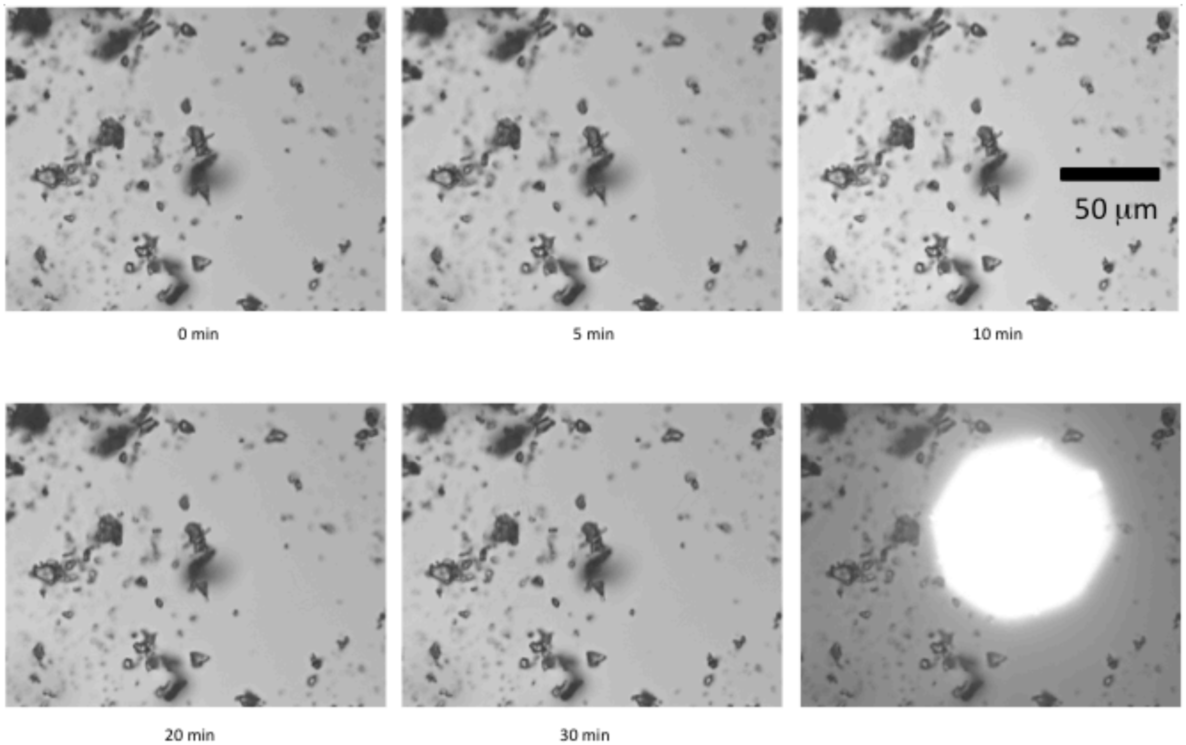

Figure S8. Bright-field microscopy images of crystals of **3** before and after UV irradiation (330-385 nm, 290 mW/cm^2) for 5, 10, 20 and 30 min at room temperature in a dry condition. The bottom-right image shows the position of the irradiated region. The calibration bar represents 50 μm.

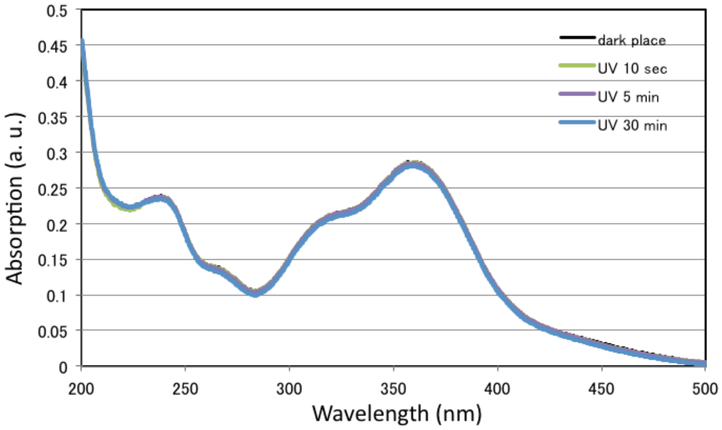

Figure S9. UV-visible spectra of **4** (10 μM) in a mixture of water and MeOH (1%). A solution of *trans*-**4** was successively exposed to 365 nm LED light (344 mW/cm^2) for 10 s, 5 min and 30 min.

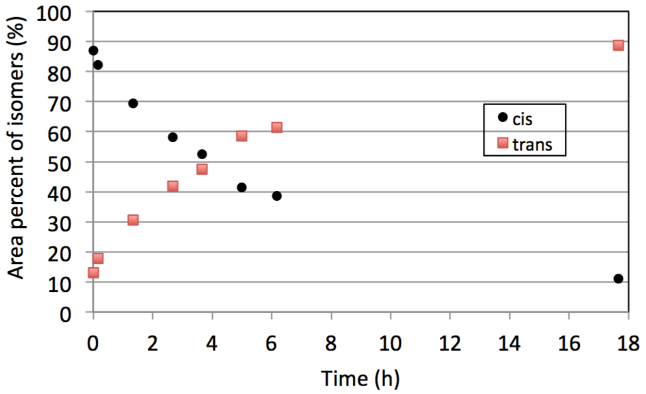

Figure S10. Thermal isomerization of *cis*-**3** at 37 ºC. 10 µM *trans*-**3** in MilliQ containing 1% MeOH was exposed to 365 nm light for 5 min, then left to stand at 37 ºC in the dark. *Cis* and *trans* isomers were analyzed by HPLC based on peak areas detected at 300 nm. $t_{1/2}$ and time constant τ were estimated as 5.6 and 8.2 hours, respectively.

Table S1. Physicochemical properties of stilbenes **1** and **2**.

Compound	Aqueous solubility[a] (μg/mL)	Melting point (ºC)	LogP_{ow}	λmax (nm)
cis-**1**	0.673	121.1	3.3	300
trans-**2**	0.0475	155.0-155.2	3.2	330

a) Aqueous solubility at 20 ºC for 24 h.

Table S2. Photoinduced isomerization of **3**

Light	Time (min)	*cis* ratio (%)	*trans* ratio (%)
visible	10	20.3	79.7
UV	10	84.8	15.2
visible	12	18.3	81.7
UV	10	86.9	13.1
visible	20	19.4	80.6

Compound **3** (10 μM) in a mixture of water and MeOH (1%) was successively exposed to white light and to a 365 nm mercury lamp (0.5 mW/cm^2). The *cis-trans* ratio was calculated from the peak areas in HPLC.

Table S3. *Cis* ratio and aqueous solubilization of **3** under UV irradiation, under visible light irradiation, and in the dark

Shake time	UV light		Visible light		Dark	
	cis ratio (%)	Solubilized **3** (μg/mL)	*cis* ratio (%)	Solubilized **3** (μg/mL)	*cis* ratio (%)	Solubility (μg/mL)
0.5 h	77	11.1			6	3.2
1 h	80	14.9	25	4.6	4	3.0
3 h	80	18.4				
5 h	75	24.7	25	5.1	7	3.5
8 h	87	24.2				

Compound **3** was shaken at 37 ºC under UV irradiation, under visible light irradiation, and in the dark. UV irradiation was performed with a mercury lamp (0.5 mW/cm^2). *Cis* ratio in the aqueous solution was evaluated from the peak areas in HPLC immediately after filtration.

Table S4. Aqueous solubilization and *cis* ratio of **3** under irradiation at various wavelengths through filters of various bandwidths.

Wavelength (nm)	Bandwidth (nm)	Aqueous solubilized **3** (μg/mL)	*Cis* ratio (%)
355	40	4.8	90
380	10	2.8	90
405	8	2.4	78
450	8	0.75	44
485	14	0.47	20
535	25	0.57	7
540	8	0.28	7

Illumination was performed with a xenon lamp using repeated cycles of irradiation and shaking (5000 flashes, then shaking for 10 sec) through filters of various bandwidths at room temperature (20-22 ºC) for 1 h. The *cis* ratio in the aqueous solution was evaluated by HPLC immediately after filtration, based on peak area.

Table S5. Aqueous solubilization of **3** under UV irradiation at various intensity levels.

Light intensity (mW/cm^2)	Aqueous solubilized **3** (μg/mL)	*cis* ratio (%)
0.00	3.0	4
4.56	12.3	89
29.0	36.2	92
290	69.6	90

Sample irradiation was conducted using 365 nm LED light (max light intensity: 290 mW/cm^2) at 37 ºC for 1 h.

Table S6. Reversibility of aqueous solubilization changes of **3** under successive UV/visible light irradiations.

Light	Aqueous solubilized **3** (fold)
visible	1.0
UV	35.5
visible	2.3
UV	37.9
visible	2.9

Sample irradiations were conducted with the white light through a filter cutting off wavelengths below 560 nm, or with 365 nm LED light (344 mW/cm^2) at 37 ºC for 1 h.

Table S7. Aqueous dissolution of UV-irradiated crystals of **3** measured in the dark, and the *cis* ratio contained in the aqueous solution

entry	Unirradiated crystals		UV-irradiated crystals	
	Aqueous solubilized **3** (µg/mL)	*cis* ratio (%)	Aqueous solubilized **3** (µg/mL)	*cis* ratio (%)
1	2.8	5	3.7	21
2	2.6	3	3.4	14
3	3.0	4	3.2	7

Crystals of **3** were irradiated with 365 nm LED light (344 mW/cm^2) at 37 ºC for 3 h. It should be noted that crystals of **3** irradiated with UV at 37 ºC for 3 h did not melt. The UV-irradiated crystals and unirradiated crystals were each shaken in phosphate buffer in the dark at 37 ºC for 1 h.

Table S8. Aqueous solubilization of **4** with or without 365 nm irradiation.

Conditions	Aqueous solubilized **4** (mg/mL)
Dark place	9.3
365 nm (0.5 mW/cm^2)	9.7
365 nm (344 mW/cm^2)	13.2

Aqueous solubilization of **4** was evaluated under 365 nm irradiation with a mercury lamp (0.5 mW/cm^2) or with an LED UV light (344 mW/cm^2), or in the dark at 37 ºC for 5 h. *Cis* isomerization was not detected by HPLC after the solubilization test; the HPLC profile of **4** contained in the aqueous solution was unchanged from the initial profile.

Table S9. Thermal isomerization of *cis*-**3** at 37 ºC detected by UV absorption measurement at 300 nm.

	HPLC area		
Time (h)	*cis*-**3**	*trans*-**3**	total
0.00	22980	4221	27201
0.17	23260	5041	28301
1.33	19040	8428	27468
2.67	15496	11131	26627
3.67	13963	12677	26640
5.00	12429	17502	29931
6.17	11633	18537	30170
17.67	3086	24581	27667
		Average	28000

10 µM *trans*-**3** in MilliQ containing 1% MeOH was exposed to 365 nm light for 5 min, then left to stand at 37 ºC in the dark. *Cis* and *trans* isomers were analyzed by HPLC based on peak areas detected at 300 nm.

Movie S1. Movie of crystals of **3** in water under UV irradiation (330-385 nm, 290 mW/cm^2) for 138 s at room temperature.

Experimental

General

3-Iodo-1-isopropyl-1*H*-pyrazolo[3,4-*d*]pirimidin-4-amine was purchased from Oakwood Products, Inc.. Melting points were determined by using a Yanagimoto hot-stage melting point apparatus and are uncorrected. ^{1}H NMR spectra were recorded on a JEOL JNM-ECA500 (500 MHz) spectrometer. ^{13}C NMR spectra were recorded on JEOL JNM-ECA500 (125 MHz) spectrometer. Chemical shifts are expressed in parts per million relative to tetramethylsilane (TMS) with coupling constants in Hz. Electrospray ionization mass spectra (ESI-MS) were recorded on a Bruker micrOTOF II spectrometer. Flash column chromatography was performed on Biotage SNAP or SNAP ultra. HPLC analyses were performed on an analytical column (GL Science Inc. Inertsil ODS-4 reversed-phase column, 5 μm, 4.6 mm x 150 mm) eluted with a mobile phase consisting of H_2O and CH_3CN at a flow rate of 1.0 mL/min, with UV monitoring at 300 or 330 nm, at 37 °C.

(*Z*)-1,2-Bis(3,4-dimethoxyphenyl)ethane (1) and (*E*)-1,2-Bis(3,4-dimethoxyphenyl)ethene (2)

To a stirred solution of 3,4-(dimethoxyphenyl)methanol (**S1**) (0.50 g, 2.98 mmol) and PPh_3 (1.87 g, 7.15 mmol) in toluene (15 mL) at room temperature was added NBS (0.58 g, 3.27 mmol). The reaction mixture was heated under reflux for 4 h, and then allowed to cool to room temperature. 3,4-Dimethoxybenzaldehyde (0.5 g, 2.98 mmol) was added, followed by K_2CO_3 (4.12 g, 29.8 mmol). Heating under reflux was resumed for a further 14 h, and then the reaction mixture was allowed to cool to room temperature. The mixture was diluted with H_2O, and extracted with AcOEt. The organic layer was dried over Na_2SO_4 and concentrated *in vacuo*. The crude solid was purified on a Biotage Isolera system (SNAP 100 g, 80% to 50% EtOAc in hexane gradient) to afford the *trans* isomer (355 mg, 40%) and the *cis* isomer (117 mg, 13%). *Cis* and *trans* isomers were identified based on the UV absorption maxima (λmax); that is, the compound possessing lower λmax was identified as **1**. The products were each recrystallized twice from a mixture of hexane and AcOEt (from 60 °C to rt) to give *trans* isomer **2** (112 mg) as colorless crystals and *cis* isomer **1** (26.1 mg) as colorless crystals. We assumed that crystals whose melting point remained the same after repeated recrystallizations represented the most stable crystal form of **1** and **2**.

***Z*-isomer 1:** Purity: 97.0% (HPLC area). ^{1}H NMR (500 MHz, $CDCl_3$) δ 6.86-6.83 (m, 4H), 6.76 (d, *J* = 8.0 Hz, 2H), 6.46 (s, 2H), 3.86 (s, 6H), 3.67 (s, 6H); ^{13}C NMR (125 MHz, $CDCl_3$) δ 149.2, 148.8, 130.8, 126.7, 119.6, 111.3, 108.6, 56.0, 55.9; m.p. 121.1 °C lit.[1] 117-118 °C; HRMS (ESI-TOF) *m/z*: $[M + H]^+$ Calcd for $C_{18}H_{20}O_4$ 301.1434; Found 304.1445.

***E*-isomer 2**: Purity: 97.9% (HPLC area). ^{1}H NMR (500 MHz, $CDCl_3$) δ 7.05 (d, *J* = 1.9 Hz, 2H), 7.03 (dd, *J* = 8.2, 1.9 Hz, 2H), 6.91 (s, 2H), 6.85 (d, *J* = 8.2 Hz, 2H), 3.94 (s, 6H), 3.90 (s, 6H); ^{13}C NMR (125 MHz, $CDCl_3$) δ 149.2, 148.8, 130.8, 126.7, 119.6, 111.3, 108.6, 56.0, 55.9; m.p. 155.2-156.0 °C lit.[1] 153-154 °C; HRMS (ESI-TOF) *m/z*: $[M + H]^+$ Calcd for $C_{18}H_{20}O_4$ 301.1434; Found 304.1482. Anal. Calcd for $C_{18}H_{20}O_4$: C, 71.98; H, 6.71. Found: C, 71.80; H, 6.86; N, 0.00.

3-Amino-1-isopropyl-1*H*-pyrazolo[3,4-*d*]pirimidin-4-amine (S3)

3-Iodo-1-isopropyl-1*H*-pyrazolo[3,4-*d*]pirimidin-4-amine (**S2**) (113 mg, 0.372 mmol) copper(I)iodide (28.3 mg, 0.149 mmol), L-proline (34.3 mg, 0.298 mmol) and K_2CO_3 (77.1 mg, 0.558 mmol) were suspended in DMSO (0.5 mL). Ammonia (aq.) solution (28%, 0.17 mL) was added to the solution, and the mixture was stirred under nitrogen for 9 h at 60 °C. The reaction mixture was diluted with water and extracted with AcOEt. The combined organic phase was dried with anhydrous sodium sulfate, filtered, and concentrated under reduced pressure. The resulting product was purified on a Biotage Isolera system (SNAP ultra 10 g, 3% to 10% MeOH in DCM gradient) to afford **S3** as a colorless solid (37.8 mg, 53%).

^{1}H NMR (500 MHz, CD_3OD) δ 8.01 (s, 1H), 4.89-4.80 (m, 1H), 1.39 (d, *J* = 6.9 Hz, 6H). MS (ESI-TOF) *m/z*: 193 $[M + H]^+$.

(*E*)-1-Isopropyl-3-(phenyldiazenyl)-1*H*-pyrazolo[3,4-*d*]pyrimidin-4-amine (3)

Amine **S3** (51.1 mg, 0.266 mmol) and nitrosobenzene (142 mg, 1.33 mmol) were dissolved in AcOH (0.5 mL) under a nitrogen atmosphere. The mixture was stirred for 10 h at 50 °C, and then further nitrosobenzene (142 mg, 1.33 mmol) was added. Stirring was continued for 23 h at 60 °C. The reaction mixture was poured into aqueous $NaHCO_3$ solution, and extracted with AcOEt. The combined organic phase was dried with anhydrous sodium sulfate, filtered, and concentrated under reduced pressure. The resulting product was purified on a Biotage Isolera system (SNAP ultra 10 g, 0% to 2.5% MeOH in DCM gradient) to afford **3** as a brown solid (48.3 mg, 65%). Compound **3** was recrystallized twice from a mixture of hexane and Et_2O (from 40 °C to rt) to give **3** as brown crystals. We assumed that crystals whose melting point remained the same after repeated recrystallizations represented the most stable crystal form of **3**.

Purity: 99.8% (HPLC area).^{1}H NMR (500 MHz, $CDCl_3$) δ 8.37 (s, 1H), 7.99-7.94 (m, 2H), 7.58-7.51 (m, 3H), 5.27 (sept, *J* = 6.6 Hz, 1H), 1.66 (d, *J* = 6.6 Hz, 6H); ^{13}C NMR (125 MHz, $CDCl_3$) δ 158.4, 157.0, 155.5,

154.6, 151.8, 132.2, 129.5, 122.9, 92.2, 49.6, 22.0.; m.p. 190-190.5 °C; HRMS (ESI-TOF) *m/z*: $[M + H]^+$ Calcd for $C_{14}H_{15}ON_7$ 282.1462; Found 282.1481.

Physicochemical properties

Photocharacterizations

The UV-visible absorption spectra of the *cis* and *trans* isomers were determined on a UV-visible spectrometer (Shimano, UV-2400PC). Sample solutions in quartz cells were irradiated with a 365 nm mercury lamp (4 W, UVP compact UV lamp, 0.5 mW/cm^2), LED UV light (3.3 W, CCS HLV-24UV365-4WNRBTNJ, 344 mW/cm^2), or white LED light (5 W, Nikki Trading Corp., NLSS05BM, 68 and 54 mW/cm^2 at 450 and 540 nm, respectively) for the indicated times at room temperature.

Aqueous solubilization

Compound **3** (about 0.2 mg) was ground and taken up in 1/15 M phosphate buffer (pH 7.4, 1.0 mL) in a 10 mL glass tube. The suspension was incubated under ultrasound irradiation for 10 min, and shaken in a temperature controllable incubator located in a dark room for the indicated time at the indicated temperature. With shaking on a plate shaker, a sample was irradiated with a 365 nm mercury lamp (4 W, UVP compact UV lamp, 0.5 mW/cm^2), 365 nm LED light (3.3 W, CCS HLV-24UV365-4WNRBTNJ, 290 mW/cm^2), or white LED light (5 W, Nikki Trading Corp., NLSS05BM, 68 and 54 mW/cm^2 at 450 and 540 nm, respectively) through a filter cutting off wavelengths below 560 nm (Atto Corp. YA-3). Irradiation was done from the top of the sample glass tube. Then, the suspension was filtered through a Millipore Millex-LG (0.20 μm).

For irradiation at various UV wavelengths, **3** (about 0.1 mg) was ground and taken up in 1/15 M phosphate buffer (pH 7.4, 0.2 mL) in a well of a 96-well white plate. Illumination over a range of wavelength from 355-540 nm was performed using a plate reader EnVision equipped with a xenon flash lamp (35 W, 500 Hz), with excitation filters of appropriate bandwidths. A protocol of irradiation and shaking cycles (5000 flashes, then shaking for 10 sec) was repeated for 1 h at room temperature (20-22 ºC). Then, the suspension was filtered through a Millipore Millex-LG (0.20 μm).

For the reversibility study, **3** (0.4 mg) was ground and taken up in 1/15 M phosphate buffer (pH 7.4, 2.0 mL) in a 10 mL glass sample tube. The suspension was incubated under ultrasound irradiation for 10 min. Then, a sample was irradiated with white LED light through a filter cutting off wavelengths below 560 nm (Atto Corp. YA-3) for 1 h at 37 ºC. Irradiation was done from the top of the sample glass tube. A portion of the suspension (200 μL) was filtered through a Millipore Millex-LG (0.20 μm), and the remaining suspension was irradiated with 365 nm LED light (3.3 W, CCS HLV-24UV365-4WNRBTNJ, 344 mW/cm^2) for 1 h. Then, a portion of the suspension (200 μL) was again filtered through a Millipore Millex-LG (0.20 μm). These operations were repeated.

The *cis*-*trans* ratio in these filtrates was immediately analyzed by HPLC with UV monitoring at 300 nm (analytical column: GL Science Inc. Inertsil ODS-4 reversed-phase column, 5 μm, 4.6 mm x 150 mm; eluent: a mobile phase consisting of H_2O and CH_3CN (40: 60); flow rate: 1.0 mL/min; temp: 37 °C). These filtration and the HPLC procedures were carried out in a typical laboratory environment where exposure to ambient light was not completely blocked. This may be the reason why a trace amount of the *cis* isomer was detected at the initial state. The remaining filtrate was diluted with DMF, irradiated with white LED light for 5 min, and injected into an HPLC with UV detection; peak areas at 300 nm were recorded. It should be noted that aqueous solutions of **3** after irradiation consisted of a mixture of *trans* form and *cis* form, and it was impossible to obtain pure *cis* solid for use as standard samples for the absolute calibration method. Therefore, the total HPLC peak areas of *trans*-**3** and *cis*-**3** at the isosbestic point (300 nm, as shown in Figure S2) were utilized for the absolute calibration method to uniformly evaluate samples with various *cis-trans* ratios. As an additional approach to uniformly evaluate samples with various *cis-trans* ratios, the filtrate was irradiated with white light for 5 minutes to obtain a photostationary state before determination of the concentration. The concentration of the sample solution was calculated using a previously determined calibration curve, corrected for the dilution factor of the sample. No degradation products were detected under any of these experimental conditions. The crystal form appeared to be unchanged after the solubilization determination, as there was no change in melting point.

Partition coefficient (LogP_{ow})

The *n*-octanol/water partition coefficient, LogP_{ow}, was determined by an HPLC method based on the OECD Guideline for Testing Chemicals 117.[2] HPLC analyses were performed on an analytical column (GL Science

Inc. Inertsil ODS-4 reversed-phase column, 5 μm, 4.6 mm x 150 mm) eluted with a mobile phase consisting of H_2O and CH_3CN (45: 55) at a flow rate of 1.0 mL/min, with UV monitoring at 300 (for **1**) or 330 (for **2**) nm, at 37 °C. The dead time t_0 was measured with thiourea as the unretained compound. Each measurement was performed in duplicate, and the mean retention time (t_R) was used for further calculations. The capacity factor of each compound, $k = ((t_R - t_0) / t_0)$, was calculated by extrapolation of the line fitted on the measured log k values of CH_3CN−aqueous mobile phase. For the reference substrates (acetanilide, methyl benzoate, toluene, 4-phenylphenol, benzyl benzoate, dibenzyl), the calculated log k values were plotted against $\log P_{ow}$ values, and the absolute calibration curve was determined ($\text{Log}P = 2.8279 \times (\log k) + 1.6035$, $R^2=0.98$). The Log$P$ values of tested compounds were calculated using a previously prepared calibration curve from the t_R values.

Optical Microscopy

We observed the azobenzene compound using an optical microscope (BX-51P, Olympus). Bright-field images were captured in the reflection mode. For investigation of the existence of the polarization plane in the sample, the transmitted polarizing microscope mode under crossed-Nicol conditions was used. For UV irradiation, a filter set (U-MWU-2, Olympus) that transmitted light with wavelengths between 335 ~ 385 nm was used to filter the light from a source (Xe, 75 W, Olympus). The filtered light was shaped into a circular beam using a diaphragm shutter and guided to the sample through an objective lens (UPLSAPO40×2, Olympus). The intensity of illuminating UV light at the sample was monitored with an optical power meter (3664, Hioki).

References

1) Barrett, T. N.; Braddock, D. C.; Monta, A.; Webb, M. R.; White, A. J. P. Total Synthesis of the Marine Metabolite (±)-Polysiphenol via Highly Regioselective Intramolecular Oxidative Coupling. *J. Nat. Prod.* 2011, **74**, 1980.

2) OECD Guidlines for Testing of Chemicals. 117. Partition Coefficient (N-Octanol/water), High Performance Liquid Chromatography (HPLC) Method. *OECD Guid. Test. Chem. 117. Partit. Coeff. (n-octanol/water), High Perform. Liq. Chromatogr. method* 2004, 1.
